# Research progress on the application of RPA-CRISPR/Cas12a in the rapid visual detection of pathogenic microorganisms

**DOI:** 10.3389/fcimb.2025.1640938

**Published:** 2025-07-30

**Authors:** Tuo Ji, Xin Fang, Yuzhi Gao, Kun Yu, Xuzhu Gao

**Affiliations:** ^1^ Lianyungang Clinical College, Bengbu Medical University and The Second People’s Hospital of Lianyungang, Lianyungang, China; ^2^ Lianyungang Clinical College, Xuzhou Medical University and The Second People’s Hospital of Lianyungang, Lianyungang, China; ^3^ Department of Central Laboratory, The Second People’s Hospital of Lianyungang City (Cancer Hospital of Lianyungang), Lianyungang, China; ^4^ Medical College, Yangzhou University, Yangzhou, China

**Keywords:** RPA, CRISPR/Cas12a, pathogenic microorganisms, point-of-care testing, visualization

## Abstract

In an increasingly complex global public health landscape, the continuous emergence of novel pathogens and the growing problem of antibiotic resistance highlight the urgent need for rapid, efficient, and precise detection technologies for pathogenic microorganisms. The innovative combination of Recombinase Polymerase Amplification (RPA) and CRISPR/Cas12a enables the rapid amplification of target gene fragments under isothermal conditions and the precise recognition and cleavage of specific nucleic acid sequences. The integration of RPA and CRISPR/Cas12a significantly enhances the sensitivity and accuracy of detection simplifies operational procedures, and reduces the dependence on specialized equipment for testing personnel. This combination demonstrates great potential for application in clinical diagnostics and point-of-care testing. This article provides a detailed overview of the principles of RPA-CRISPR/Cas12a and its latest research progress in the field of pathogen detection, aiming to promote the widespread application of RPA-CRISPR/Cas12a technology in clinical medicine and public health and to offer theoretical support for its further optimization.

## 1 Introduction

Pathogenic microorganisms can cause infectious diseases, chronic diseases, and immune system disorders, and in severe cases, even lead to death. Therefore, immediate and accurate detection and treatment of pathogenic microorganisms are crucial for maintaining human life and health. Current standard detection techniques for pathogenic microorganisms include traditional culture methods, next-generation high-throughput sequencing, and other molecular biology techniques. However, these detection methods have their respective limitations. Traditional pathogenic microorganism culture techniques are time-consuming, requiring days to obtain results, which cannot meet the needs of Point-of-Care Testing (POCT). Polymerase chain reaction (PCR) has high requirements for laboratory technicians, equipment, and operating environments, making it impossible to detect outside the laboratory (Alsharksi et al., 2024). In recent years, detection methods combining isothermal amplification technology with CRISPR/Cas systems have gradually emerged, making up for the shortcomings of current pathogenic microorganism detection.

Recombinase Polymerase Amplification (RPA) is an isothermal nucleic acid amplification technology that can initiate the amplification process by the synergistic action of recombinase, single-strand binding protein, and DNA polymerase at a constant temperature of approximately 37°C. The RPA reaction is rapid, completing amplification in only 15–30 minutes. Due to the absence of complex temperature control equipment and its simple operation, RPA technology is suitable for on-site rapid detection (Lobato and O’Sullivan, 2018).

CRISPR is a natural immune system derived from bacteria and archaea. The CRISPR system recognizes and cleaves foreign invading nucleic acids through RNA-guided nucleases, protecting host cells from pathogen infection. In recent years, scientists have modified and optimized CRISPR technology, leading to its widespread application in multiple fields, such as gene editing, functional genomics research, and pathogen detection (Cong et al., 2013). Cas proteins are a class of proteins associated with the CRISPR system, widely present in archaea and bacteria, and serve as the core component of their adaptive immune system. The CRISPR/Cas12a system is a gene editing and detection technology based on CRISPR and the Cas12a protein, belonging to the class 2 and type V CRISPR system. Cas12a is a CRISPR-RNA (crRNA) guided nuclease that can specifically recognize and cleave target DNA containing a Protospacer Adjacent Motif (PAM) sequence (Ma et al., 2022).

Leveraging the advantages of RPA and CRISPR/Cas12a, Jennifer Doudna’s team at the University of California developed the DETECTR (Democratic and Exclusive Targeting of Exogenous Cytoplasmic RNA) system (He et al., 2024). This system combines the efficient isothermal amplification of RPA with the specific nucleic acid cleavage ability of CRISPR/Cas12a, making it particularly suitable for real-time and sensitive pathogen detection with broad application prospects.

Currently, RPA-CRISPR/Cas12a technology has been applied to the detection of pathogenic microorganisms such as human papillomavirus, *Staphylococcus aureus*, and *Plasmodium*. This article reviews the principles of RPA-CRISPR/Cas12a technology and its research progress in pathogenic microorganism detection, aiming to provide references for the further development and application of this technology.

## 2 Basic principles of RPA

Isothermal nucleic acid amplification technologies such as RPA, Loop-Mediated Isothermal Amplification (LAMP), and Rolling Circle Amplification (RCA) can exponentially amplify specific DNA target sequences using different reaction systems at constant temperatures. As an emerging isothermal nucleic acid amplification technology, RPA can rapidly amplify target nucleic acids within 10–30 minutes through the synergistic action of multiple enzymes and proteins at 37-42°C (Munawar, 2022). The RPA reaction system primarily includes three enzymes: a recombinase that binds to single-stranded nucleic acids, a single-stranded DNA-binding protein, and a strand-displacing DNA polymerase. In the presence of ATP, the recombinase binds to oligonucleotide primers to form a protein-DNA complex, which can recognize homologous sequences in double-stranded DNA, invade the double-stranded DNA after accurate positioning, and initiate strand displacement reactions. Subsequently, single-stranded DNA-binding proteins rapidly bind to the displaced single-stranded DNA to prevent reannealing. Meanwhile, DNA polymerase extends from the primer. The other strand in the double strand maintains a stable single-stranded structure under the action of single-stranded DNA-binding proteins. With the extension of DNA polymerase, the two parental strands gradually separate, eventually forming two new double-stranded DNAs. This process repeats, achieving exponential amplification of target DNA quickly (Daher et al., 2016). **Table 1** summarizes a comparison of RPA with other nucleic acid amplification technologies.

**Table 1:** Comparison of RPA and other amplification techniques.

Comparison	RPA	PCR	RCA	LAMP
Tool enzymes	Recombinase, single-strand binding protein, DNA polymerase	Helicase, Taq DNA polymerase, recombinase	Phi29 DNA polymerase	Bst DNA polymerase
Reaction Time	10–30 min	1–2 h	2 h	15–60 min
Sensitivity	1–100 copies/μL	10–100 copies/μL	1 copies/μL	1–100 copies/μL
Specificity	High	High	Extremely high	Extremely high
Reaction temperature	37-42°C	95°C-55°C	30-37°C	60-65°C
Primer Design	2 (30–35 bp)	2 (18–25 bp)	1-2 (15–30 bp)	4-6 (15–25 bp)
Equipment cost	low	high	low	Medium
Product detection methods	Fluorescent signaling, lateral flow strips, agarose gel electrophoresis	Agarose gel electrophoresis, fluorescent probes	Agarose gel electrophoresis, fluorescent probes	Turbidity measurement, fluorescent dyes (SYBR Green, TaqMan), visual colorimetry (pH indicators)
Advantages	Fast reaction speed, easy operation, high portability	Strong specificity, high sensitivity, wide range of applications	High specificity and sensitivity	High specificity and rapid detection
Shortcomings	High requirement for primer design; limited detection throughput	High equipment requirements, complex operation, time-consuming	High template preparation requirements, high testing costs, complex data analysis	Complex primer design, potential for non-specific products

## 3 Principles of the CRISPR/Cas system

The CRISPR system consists of repeated DNA fragments and spacer sequences. These spacer sequences are typically derived from foreign DNA. When a virus invades a microorganism, its CRISPR system can record its genetic information and integrate it into the CRISPR region of microorganism genome. These spacers and repeat sequences are alternately arranged to form part of the CRISPR locus. When the same foreign genetic material invades again, the CRISPR/Cas system uses these spacer sequences to recognize and precisely cleave the target DNA, providing immune protection. Cas genes refer to CRISPR-associated genes that encode Cas proteins. Cas proteins have helicase activity, capable of independently integrating the genetic information of foreign invaders into spacer sequences and synergistically degrading invading nucleic acid fragments with high efficiency and specificity in the CRISPR region (Manghwar et al., 2019).

CRISPR-Cas systems are divided into two major categories, further classified into multiple types (Type I to Type VI) and 33 subtypes. Among them, Class 1 (Type I, III, IV) uses multiple Cas proteins in their CRISPR ribonucleoprotein effector nucleases, while Class 2 systems (Type II, V, VI) use a single Cas protein (Koonin et al., 2017). CRISPR/Cas12a (Cpf1) belongs to Type V in Class 2 and is a single-stranded RNA-guided gene editing tool (Zetsche et al., 2015). In 2015, Zetsche et al. screened 16 Cas12a proteins from 46 Cas12a family proteins through bioinformatics analysis and experimental verification for PAM sequence determination and functional analysis. They found Cas12a proteins derived from *Francisella novicida U112*, *Acidaminococcus* sp. *BV3L6*, *Lachnospiraceae bacterium ND2006*, *Candidatus Methanoplasma termitum*, and *Moraxella bovoculi* can efficiently perform double-strand cleavage of target DNA (Swarts and Jinek, 2019). Cas12a uses guide RNA (CRISPR RNA, crRNA) to search for matching or complementary nucleic acid sequences. crRNA is one of the key components in the CRISPR/Cas12a system, functioning to recognize and bind to specific target DNA sequences, thereby activating the nuclease activity of the Cas12a protein. Cas12a protein recognizes and cleaves DNA, which relies on a specific PAM sequence near the target sequence called cis-cleavage. However, the uniqueness of Cas12a lies in its trans-cleavage activity: once activated, it can non-specifically cleave single-stranded DNA (ssDNA) throughout the reaction system, and this process does not depend on the presence of a PAM sequence (Daneshgar et al., 2025). Subsequently, the cell causes gene editing or mutation through its repair mechanisms. This trans-cleavage activity makes Cas12a of great application value in nucleic acid detection and molecular diagnostics.

## 4 RPA-CRISPR/Cas12a reaction system

**Figure 1:**
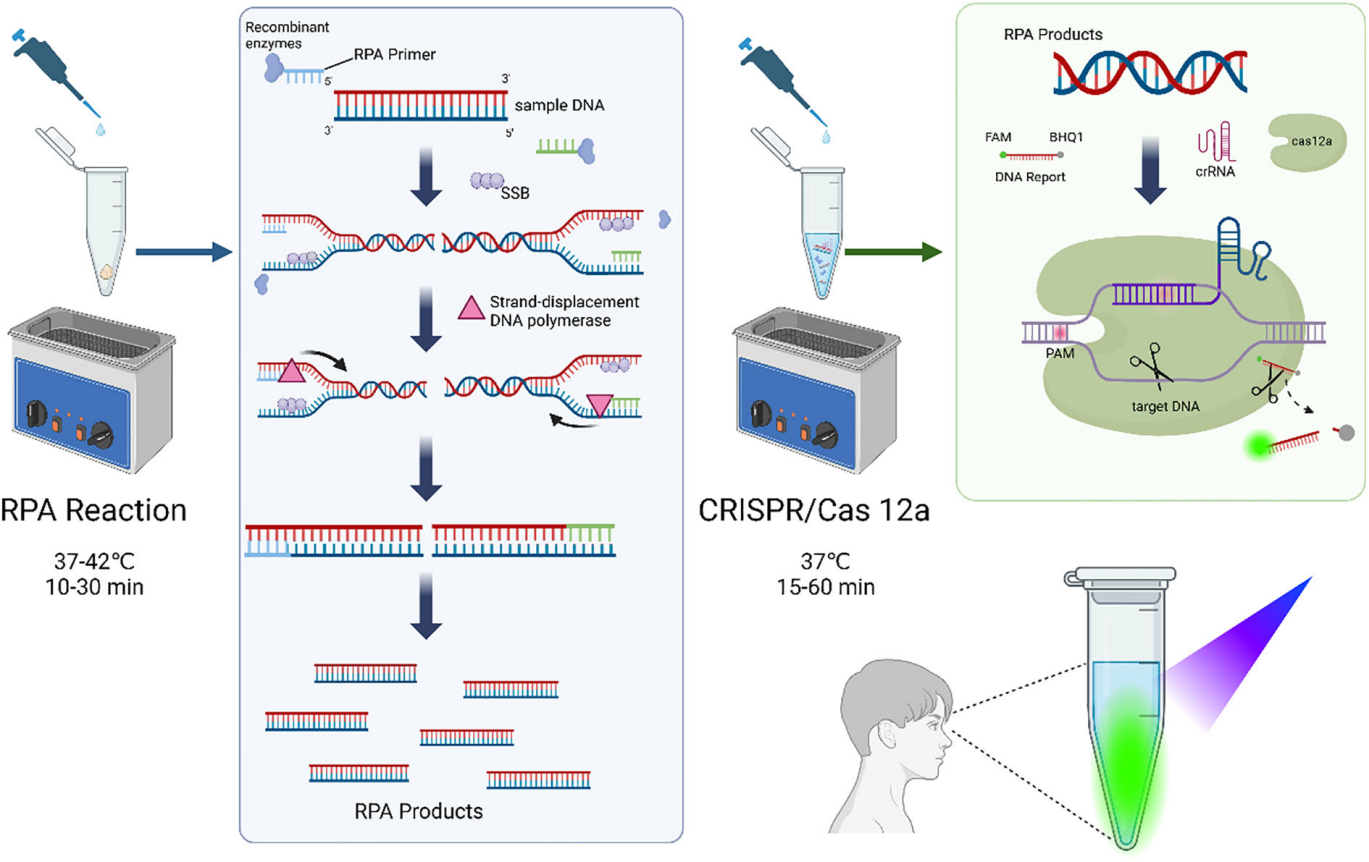
Principle of RPA-CRISPR/Cas12a. Created in BioRender. Ji, (2025) https://BioRender.com/au4gcl0.

Based on in-depth research on RPA and CRISPR/Cas systems, multiple research teams have proposed combining RPA technology with the CRISPR/Cas12a system for pathogenic microorganism detection. This combined technology offers unique advantages, not only avoiding the dependence of traditional PCR on complex temperature control equipment but also achieving specific and highly sensitive detection of target sequences. The RPA-CRISPR/Cas12a detection system integrates the isothermal amplification capability of recombinase polymerase amplification with the highly specific nucleic acid cleavage function of the CRISPR/Cas12a system. In this system, RPA can rapidly amplify target nucleic acids at a constant temperature of 37-42°C, generating large amounts of DNA products. Subsequently, the CRISPR/Cas12a system recognizes and binds to the target DNA through crRNA, activating the trans-cleavage activity of Cas12a to non-specifically cleave fluorescent reporter molecules (single-stranded DNA), producing detectable signals. The combined action of both systems enables the detection of pathogenic microorganisms at an optimal temperature of approximately 37°C (Senthilnathan et al., 2023). The unique design of the RPA-CRISPR/Cas12a system makes it an efficient and sensitive detection platform suitable for rapid identification and quantitative analysis of specific nucleic acid sequences. The quantitative analysis is achieved through monitoring the generated fluorescent signals, with two detection methods employed. The first method involves quantitatively analyzing the fluorescence intensity in the CRISPR detection chamber excited by blue light in a gel cutter using ImageJ software (Zhang et al., 2025). The second method uses a quantitative fluorescence PCR instrument to read fluorescence values (Cai et al., 2025). As the intensity of the fluorescent signal is proportional to the concentration of target nucleic acids, quantitative analysis can be performed via standard curves generated from serial dilutions of known target templates. This approach ensures precise correlation between signal strength and nucleic acid concentration, enabling reliable quantification in the assay. Through this optimized combination, the system can provide powerful detection capabilities at the molecular level, which is widely applied in biomedical research and clinical diagnostics (Xu et al., 2022). The principle of RPA-CRISPR/Cas12a assay is shown in **Figure 1**.

The RPA reaction is performed first. Recombinase proteins bind to primers to form DNA nucleoprotein microfilaments, which can quickly locate the target gene fragment, and the primers bind tightly to their homologous sequences, followed by an amplification reaction of the target gene fragment under the action of DNA polymerase. During this process, single-stranded binding proteins bind to the displaced DNA single strand, preventing the single strand from reverting to a double strand. Repeating this process exponentially amplifies the target gene. The CRISPR/Cas12a complex and DNA fluorescent reporter probe are subsequently added to the reaction tube. crRNA recognizes the target DNA and activates Cas12a trans-cutting activity to cleave the DNA probe non-specifically. Finally, the fluorescence signal is observed using a portable fluorescence irradiator.

## 5 Steps of the RPA-CRISPR/Cas12a reaction

### 5.1 Design and screening of RPA primers

The software commonly used for designing RPA primers includes Primer Premier, Primer Express, and Oligo, among others. The primer screening steps for RPA mainly include selecting the target region, designing primers, and determining the optimal primers. RPA primer design must follow the following basic principles (Higgins et al., 2019): Primer length should be between 30–35 nucleotides. This length ensures effective recombinase binding while avoiding reduced activity due to excessively short primers or the formation of intramolecular secondary structures due to excessively long primers. The 5’ end of the primer should preferably use cytosine-rich sequences to reduce the formation of polyguanine structures at the 5’ end, thereby minimizing non-specific binding. The 3’ end of the primer should use a combination of guanine and cytosine to enhance the binding force between the primer and polymerase, improving amplification stability. Primers should avoid using continuous polypurine or polypyrimidine sequences to prevent unstable primer structures or non-specific binding. Additionally, the length of the amplified DNA fragment should be between 70–500 bp to ensure amplification efficiency. Based on these design principles, primer performance in RPA reactions can be optimized while reducing non-target sequence amplification and improving experimental result accuracy. RPA primer verification is a critical step to ensure amplification efficiency and specificity, which can be accessed via electrophoresis analysis for primer specificity. After performing RPA amplification with designed primers, agarose gel electrophoresis is used to observe whether the amplification products exhibit the expected specific bands. If the band position matches the target product and no other non-specific bands appear, it indicates good primer specificity.

### 5.2 Establishment of the CRISPR/Cas12a system

The key step in establishing the CRISPR/Cas12a system is crRNA design, which must ensure specific binding to the target sequence and avoid non-specific cleavage (Mao et al., 2024). After screening the optimal RPA primers, CRISPR online tools (such as CHOPCHOP and CRISPR Recognition Tool) can be used to design corresponding crRNAs. The DNA products amplified by RPA provide an accurate template for constructing the crRNA library.

### 5.3 Establishment of the RPA-CRISPR/Cas12a detection method

First, samples are obtained using strict operating procedures and standards according to the detection purpose, and nucleic acids are extracted from the samples using appropriate methods. The extracted nucleic acids are added to a reaction tube, along with RPA primers, enzymes, and other relevant components. RPA amplification of the target nucleic acid sequence is performed at an appropriate temperature (37-42°C). Sufficient nucleic acid copy templates are generated through isothermal amplification for recognition by the CRISPR/Cas12a system. The amplified nucleic acid template is mixed with the CRISPR/Cas12a system, and relevant reaction components for the CRISPR/Cas system are added. crRNA recognizes and binds to the target sequence, and the Cas12a protein specifically recognizes and binds to the DNA, forming a ribonucleoprotein interference complex. Thus, the RPA-CRISPR/Cas12a complex is constructed. After the reaction is complete, result reading is required. In most studies, the detection results of the RPA-CRISPR/Cas12a detection system are read through the generation of fluorescent signals. After the Cas12a protein recognizes and binds to the target DNA under the guidance of crRNA, its trans-cleavage activity is activated, enabling non-specific cleavage of fluorescently labeled probes added to the reaction system. When excited by an appropriate light source, specific fluorescent signals from the probes can be observed, and the presence or absence of fluorescent signals determines the detection result (Deltcheva et al., 2011). Additionally, combining the RPA-CRISPR/Cas12a process with lateral flow strips (LFS) can be used to quickly visualize reaction results (Hsiung et al., 2025). Lateral flow strips employ nitrocellulose membranes with pre-coated detection lines (anti-fluorescent dye antibodies, e.g., anti-FAM) and control lines (anti-biotin antibodies). In negative samples, the single-stranded DNA probe remains uncleaved. As the reaction reagent migrates to the control area, the biotin moiety at the probe’s terminus is captured by streptavidin-conjugated gold nanoparticles (AuNPs), inducing a visible chromatographic change through nanoparticle aggregation. When the mixture containing CRISPR-Cas12a cleavage products flows through the strip, fluorescently labeled reporter oligonucleotides bind to the detection line, generating a distinct colorimetric signal that enables naked-eye interpretation (Jiang et al., 2023). Because of its rapidity and convenience, LFS has attracted more attention in POCT.

## 6 Applications of RPA-CRISPR/Cas12a technology

RPA-CRISPR/Cas12a technology has been widely applied in the detection of pathogenic microorganisms such as bacteria, fungi, virus and parasites due to its advantages of rapidity, strong specificity, low equipment dependency, simple operation, and visualizable results. Notably, Yongming Wang pioneered the integration of RPA with CRISPR/Cas12a technology in 2018, demonstrating for the first time that this technique enables highly sensitive detection of mycoplasmas (Wang et al., 2019). This work not only establishes the technical foundation for the rapid detection of pathogenic microorganisms but also accelerates its translational application in critical areas.

### 6.1 Applications in bacterial detection

RPA-CRISPR/Cas12a technology is mainly applied to the detection of bacteria such as *Mycobacterium tuberculosis*, *Escherichia coli*, *Haemophilus parasuis*, *Brucella*, *Shigella*, *Salmonella*, and *Staphylococcus aureus* (**Table 2**), and is widely used in the fields of healthcare, food safety and environmental monitoring (Xue et al., 2025).

**Table 2:** Application of RPA-CRISPR/Cas12a technology in bacterial detection.

Bacteria	Target gene	Reaction temperature	Reaction time	LOD	Reference
*Haemophilus parasuis*	Omp P2	37°C	50 min	0.163 pg/μL DNA	(Zhang et al., 2023)
*Escherichia coli*	rfbE	37°C	20-30min	1 CFU/mL	(Wang et al., 2020)
*Mycobacterium tuberculosis*	IS6110	37°C	60 min	50 CFU/mL	(Xiao et al., 2023)
*Salmonella*	InvA mcr-1	37-42°C	40 min	1 CFU/mL	(Fu et al., 2022)
*Staphylococcus aureus*	clfA	37°C	30 min	5 copies/μL	(Liu et al., 2023b)
*Helicobacter pylori*	vacA	37°C	45 min	1.4 copies/µL	(Li et al., 2023b)
*Neisseria gonorrhoeae*	por A	39°C、37°C	60 min	5 pg/μL	(Tu et al., 2023)
*Leptospira*	lipL32	39°C	75 min	100 cells/mL	(Jirawannaporn et al., 2023)
*Pseudomonas aeruginosa*	oprL	39°C、37°C	30 min	60 fp DNA	(Liu et al., 2023a)
*Brucella*	bp26	39°C、42°C	35 min	10 copies/μL	(Dang et al., 2023)
*Bacillus cereus*	nheA、nheB、nheC、hblA	37°C	55 min	10 copies/μL	(Li et al., 2024)

*Mycobacterium tuberculosis* (MTBC) is a Gram-positive bacterium that primarily affects the lungs but can also invade other parts of the body (Huang et al., 2021). Xiao applied isothermal amplification technology to the conserved genes S6110 and IS1081 of MTBC, reacted the RPA-amplified products with CRISPR/Cas12a protein and designed crRNA, completed the entire process from sample to result in approximately 1 hour with final results visualized using blue light. The results demonstrated 100% specificity in clinical isolates (Compiro et al., 2025).

*Escherichia coli* (*E*. *coli*) is a Gram-negative bacterium commonly found in the intestines of humans and other warm-blooded animals. Pathogenic *E*. *coli* can cause symptoms such as diarrhea, abdominal cramps, nausea, vomiting, and fever. Clinical identification of *E*. *coli* typically relies on laboratory culture, observing bacterial morphology, biochemical reactions, and growth characteristics, which requires 48–72 hours and specialized equipment (Figler and Dudley, 2016). Using RPA-CRISPR/Cas12a technology, the detection process can be completed in approximately 40 minutes, with high sensitivity, a limit of detection (LOD) of 2.4 CFU/mL for *E*. *coli*, and reduced risk of cross-reactions and false-positive results due to the technology’s highly specific target recognition.

### 6.2 Applications in fungal detection

RPA-CRISPR/Cas12a technology has demonstrated significant application potential in fungal detection, although its current application in fungal detection is limited due to incomplete genomic information of fungi. It has been reported that this technology can detect *Phytophthora sojae* within one hour, with a detection limit as low as 10 pg/μL of genomic DNA, no cross-reactions with other closely related species, and visualizable results, making it suitable for on-site rapid detection and use in resource-constrained areas (Guo et al., 2023).

### 6.3 Applications in virus detection

RPA-CRISPR/Cas12a holds broad application prospects in viral nucleic acid detection (**Table 3**). The recent study by Liu et al. (2024) established an RPA-CRISPR/Cas12a detection system based on the conserved sequences of the HPV genome using the concept of “universal primers,” enabling multiplex nucleic acid detection of HPV. This detection system requires only 45 minutes from sample addition to result reading, with a detection sensitivity as high as 1 copy/μL for each high-risk type. Dan (Xiong et al., 2020) established an RPA-CRISPR/Cas12a system based on the conserved regions of coronavirus nucleic acid sequences ORF1ab and N gene, designed and screened specific primers. The RPA-CRISPR/Cas12a detection exhibits single-copy sensitivity, and its detection sensitivity is unaffected by other viral DNAs. The advantage of CRISPR/Cas12a technology in combination with RPA is that it can be tested in the field without the need for expensive tools or trained personnel, making it particularly suitable for use in resource-constrained field environments. The results showed 100% consistency with the RT-qPCR for the negative and positive samples.

**Table 3:** Application of RPA-CRISPR/Cas12a technology in virus detection.

Virus	Target gene	Reaction temperature	Reaction time	LOD	Reference
African swine fever virus	ASFV	37°C	40 min	5.8 copies/μL	(Mao et al., 2023)
Human papillomavirus	L1、E6/7	37°C	45 min	1 copy/μL	(Liu et al., 2024)
SARS-CoV-2	ORF1ab、N	37°C	50 min	1 copy/μL	(Xiong et al., 2020)
Mouse Poxvirus	D6R、E9L、N3R	37°C	30 min	1 copy/μL	(Zhao et al., 2023)
Severe fever with thrombocytopenia syndrome virus	L	37°C	60 min	1 copy/μL	(Huang et al., 2022)
Respiratory syncytial virus	RSV A RSV B	37°C	65 min	1.38×10^1^ copies/μL	(Gong et al., 2022)
Monkeypox Virus	F3L B6R	37°C	40 min	100 copy/μL	(Li et al., 2023a)

### 6.4 Applications in parasite detection

Currently, parasitic infections remain a significant public health issue globally, particularly in regions with scarce medical resources and harsh environmental conditions. Infections caused by Toxoplasma gondii, Trichomonas vaginalis, Cryptosporidium parvum, etc., are common. RPA-CRISPR/Cas12a technology has demonstrated remarkable advantages in parasite detection (**Table 4**), capable of detecting conserved gene sequences of specific parasites to determine infections within a short time, facilitating subsequent clinical treatment. Huagui Wei (Wei et al., 2023) developed an RPA-based detection method that achieves highly sensitive detection of Plasmodium DNA through signal amplification and conversion by the CRISPR/Cas12a system. This method uses fluorescence detection, lateral flow strips, or naked-eye observation to determine the presence of Plasmodium genus. The detection limit of this method is 1 copy of plasmid DNA per microliter, with no cross-reactions with other pathogen DNAs in blood, realizing the application of RPA-CRISPR/Cas12a technology in rapid parasite detection.

**Table 4:** Application of RPA-CRISPR/Cas12a technology in parasite detection.

Parasite	Target gene	Reaction temperature	Reaction time	LOD	Reference
*Toxoplasma*	B1	37°C	55 min	3.3 copies/μL	(Sun et al., 2024)
*Trichomonas vaginalis*	Actin	37°C	60 min	1 copies/μL	(Li et al., 2022)
*Plasmodium*	SSRRNA	37°C	40 min	1 copies/μL	(Wei et al., 2023)
*Neospora caninum*	Nc5	37°C	90 min	1 parasite/mL	(Wang et al., 2024)
*Clonorchis sinensis*	NAD1	37°C	40 min	0.1 ng/μL	(Phuphisut et al., 2024)

## 7 Discussion

Rapid diagnosis of pathogenic microorganisms is essential for guiding medication use and alleviating the disease. Conventional pathogenic microorganism detection methods are time-consuming, labor-intensive, and unsuitable for immediate on-site detection. While the development of isothermal amplification technology further promotes the development of immediate on-site detection technology. RPA, as a highly efficient means of isothermal nucleic acid amplification, has already demonstrated its unique advantages and prospects for broad application in pathogenic microorganism detection. In the past, the commonly used detection methods for RPA amplification products include nucleic acid gel electrophoresis, real-time fluorescence, and lateral flow strips. LFS is a paper-based POCT device consisting of a sample pad, a binding pad, a nitrocellulose membrane, and a water-absorbent pad. The most commonly used signal labels for lateral flow strips are colloidal gold nanoparticles and 100 nm spherical gold nanoparticles based on the material’s signal amplification strategy due to their high molar extinction coefficient and light intensity. Combining the LFS of encapsulated gold nanoparticles with RPA technology for visual detection of amplification products, positive signals can be observed on test strips with the naked eye in a few minutes, significantly shortening the detection time and simplifying the operation steps. The RPA-LFS technology is widely used for the rapid detection of pathogenic microorganisms such as *Staphylococcus haemolyticus* (Ji et al., 2023), *Staphylococcus epidermidis* (Wang et al., 2023), *Acinetobacter baumannii* (Wang et al., 2022b), *Streptococcus pneumoniae* (Wang et al., 2022a), and so on. However, the potential generation of primer dimers and the low sensitivity of RPA-LFS have limited its application in quantitative detection and detection of low-load pathogens.

RPA-CRISPR/Cas12a technology, an emerging pathogen detection technology, cleverly combines the highly efficient and sensitive nucleic acid amplification capability of RPA technology with the specific DNA recognition and trans-cutting function of the CRISPR/Cas12a system to achieve highly sensitive molecular detection. RPA-CRISPR/Cas12a not only improves the efficiency of nucleic acid detection but also significantly broadens the scope of application of RPA technology, showing broad application prospects in on-site diagnosis of pathogenic microorganisms and screening of drug-resistant genes.

The RPA-CRISPR/Cas12a system is rapidly becoming an efficient tool for clinical detection. However, this technology also has its limitations and challenges. For example, the limitation in multiplex detection capability, where the optimization and standardization of multiplex detection remain a challenge. The research team adopted a novel microfluidic chip technology that combines CRISPR/Cas12a gene editing tools with RPA technology through a “ Microfluidic space coding “ strategy to construct a MiCaR multi-target nucleic acid detection platform. This detection system can identify and distinguish up to 30 different pathogens within 40 minutes, significantly enhancing detection sensitivity and specificity, and pioneering a new protocol for nucleic acid detection methods (Xu et al., 2022). Aerosol contamination is also a common issue in the application of RPA-CRISPR/Cas12a technology, which may originate from sample processing and nucleic acid amplification steps and can occur at various stages of the experiment. In recent years, many studies have employed single-tube RPA-CRISPR/Cas12a detection methods (Liu et al., 2023b), allowing RPA reactions and the CRISPR/Cas12a system to occur in a single tube, avoiding aerosol contamination caused by opening the lid. Currently, there are two main methods to achieve single-tube detection: one is adding the CRISPR/Cas12a reaction system to the tube lid, allowing the system on the lid to fall into the completed RPA reaction system in the tube after RPA completion to finish the entire reaction (Wang et al., 2019); the other uses double-layer reaction tubes, with the inner tube bottom containing small holes and loaded with the CRISPR/Cas12a reaction system, while the outer tube contains the RPA reaction system. After the outer tube reaction is complete, centrifugation or other means are used to transfer the inner tube reaction solution into the outer tube. Finally, results are read using appropriate methods based on the selected indicator. These one-step technologies integrate RPA and CRISPR detection into a single tube, offering simple operation and avoiding aerosol contamination (Hu et al., 2022). Furthermore, the issues of false positives and false negatives cannot be overlooked. The relatively low reaction temperature and high concentrations tends to induce the formation of non-specific amplification products and primer dimers (Mao et al., 2022). Alternatively, off-target of crRNA in the CRISPR/Cas12a system may trigger erroneous cleavage signals, which can lead to false-positive results. However, the integration of RPA and CRISPR/Cas12a can not only achieve a marked reduction in the incidence of false-positive outcomes but also enhance the specificity and efficiency of detection (Xue et al., 2025). False-negative results may arise from the presence of inhibitors in samples that impair enzyme activity or from the failure to effectively amplify and recognize low-abundance targets. To address these concerns, improvements can be made through strategies such as optimizing primer design and strictly controlling reaction conditions. For example, using bioinformatics tools to assess secondary structures and binding free energy. In addition, researches have found that the addition of DMSO and betaine can enhance the effects of Mg^2+^ and ATP binding during amplification and prevent primer dimer formation (Moon et al., 2022; Kim et al., 2023). At the same time, the cost of CRISPR/Cas12a-related reagents (e.g., Cas12a proteins, customized crRNAs) is high relative to commercially available PCR reagents. However, CRISPR/Cas12a does not need to rely on expensive temperature-controlled instrumentation and has a significant advantage in terms of equipment investment, creating a unique application balance of ‘slightly higher reagent cost but lower equipment threshold.’ In the future, the reaction cost can be reduced by optimizing the expression and purification process of Cas12a protein, improving the synthesis technology of crRNA, and optimizing the reaction system, thereby facilitating the promotion of its application.

With continuous technological advancements, the deep integration of the CRISPR/Cas12a system with traditional detection technologies demonstrates enormous potential to revolutionize *in vitro* diagnostics. This innovative detection strategy not only boasts high sensitivity and specificity but also provides new possibilities for the future development of clinical laboratory techniques due to its simple operation and rapid result generation. Its broad application prospects in pathogen detection, cancer screening, and genetic disease diagnosis indicate that it will become the core force of next-generation diagnostic technologies, driving medical detection toward greater efficiency and precision.
